# Inhibitory effect of napabucasin on arbidol metabolism and its mechanism research

**DOI:** 10.3389/fphar.2023.1292354

**Published:** 2023-11-28

**Authors:** Jingjing Nie, Hailun Xia, Ya-Nan Liu, Yige Yu, Ren-Ai Xu

**Affiliations:** ^1^ Department of Pharmacy, The Third Affiliated Hospital of Wenzhou Medical University, Wenzhou, Zhejiang, China; ^2^ Department of Pharmacy, The First Affiliated Hospital of Wenzhou Medical University, Wenzhou, Zhejiang, China; ^3^ Key Laboratory of Diagnosis and Treatment of Severe Hepato-Pancreatic Diseases of Zhejiang Province, The First Affiliated Hospital of Wenzhou Medical University, Wenzhou, Zhejiang, China

**Keywords:** arbidol, napabucasin, drug–drug interactions, metabolism, CYP3A4

## Abstract

As a broad-spectrum antiviral, and especially as a popular drug for treating coronavirus disease 2019 (COVID‐19) today, arbidol often involves drug–drug interactions (DDI) when treating critical patients. This study established a rapid and effective ultra-performance liquid chromatography–tandem mass spectrometry (UPLC-MS/MS) method to detect arbidol and its metabolite arbidol sulfoxide (M6-1) levels *in vivo* and *in vitro*. In this study, a 200 μL incubation system was used to study the inhibitory effect of the antitumor drug napabucasin on arbidol *in vitro*, with IC_50_ values of 2.25, 3.91, and 67.79 μM in rat liver microsomes (RLMs), human liver microsomes (HLMs), and CYP3A4.1, respectively. In addition, we found that the mechanism of inhibition was non-competitive inhibition in RLM and mixed inhibition in HLM. In pharmacokinetic experiments, it was observed that after gavage administration of 48 mg/kg napabucasin and 20 mg/kg arbidol, napabucasin inhibited the metabolism of arbidol *in vivo* and significantly changed the pharmacokinetic parameters of arbidol, such as AUC_(0-t)_ and AUC_(0-∞)_, in rats. We also found that napabucasin increased the AUC_(0-t)_ and AUC_(0-∞)_ of M6-1, the main metabolite of arbidol. This study provides a reference for the combined use of napabucasin and arbidol in clinical practice.

## 1 Introduction

In 1993, arbidol was introduced as a broad-spectrum antiviral drug to prevent and treat influenza infection (Boriskin et al., 2008). Arbidol 200 mg taken three times a day for about a week can reduce the duration of influenza by 1.7–2.65 days in clinical trials (Leneva et al., 2009). In recent years, coronavirus disease 2019 (COVID‐19) has posed a significant threat to respiratory diseases and lung infections (Kirtipal et al., 2020; Vellingiri et al., 2020). Arbidol has been widely used for the prevention of COVID-19 and to improve the control of severe acute respiratory syndrome coronavirus 2 (SARS-CoV-2) infection (Nguyen et al., 2021; Zhou et al., 2021). Other applications of arbidol can be found as well. For example, studies showed that arbidol was also an effective and safe way to treat verruca plantaris and that topical administration of arbidol was more acceptable to patients (Chen et al., 2020).

COVID-19 symptoms are commonly treated with combinations of drugs, but they can also be harmful. For example, the combination of lopinavir/ritonavir (LPV/r) and arbidol will be a risk factor for liver injury in patients with non-critical COVID-19 (Cai et al., 2020). Studies reported that when LPV/r was combined with arbidol, the C_max_ of arbidol was increased and the AUC_(0-∞)_ of arbidol was significantly increased from 705.6 to 1250.3 ng/mL*h (Huang et al., 2021). In addition, pharmacokinetic and metabolic differences caused by administration of arbidol were found in male and female rats. When arbidol was used in combination with the famous Chinese medicine Lianhua Qingwen to treat COVID-19 (Liu et al., 2021), the levels of eosinophils and lymphocytes in patients were increased, which indicated that the combination was helpful in treating the progression of COVID-19.

In cancer treatment, napabucasin is a therapy that targets signal transducer and activator of transcription 3 (STAT3) pathways (Hubbard and Grothey, 2017; Chang et al., 2019). Several types of cancer can be treated with napabucasin alone or in combination, including advanced colorectal cancer, pancreatic cancer, and squamous cell carcinoma (Hubbard and Grothey, 2017). Furthermore, napabucasin administration in rats inhibits both apoptosis and oxidative stress, protecting neonatal rat neuronal cells from damage (Wang et al., 2019). There is a possibility that napabucasin could be used in the future to treat brain damage.

Drugs are often used in combination during the treatment of cancer, as cancer patients were also found to be affected with COVID-19 (Liu et al., 2020; Brito-Dellan et al., 2022). However, drug–drug interactions (DDI) between napabucasin and the antiviral drug arbidol have not been reported previously. Therefore, rapid detection of plasma levels of arbidol and its metabolite was established by using ultra-performance liquid chromatography–tandem mass spectrometry (UPLC-MS/MS), and further studies of its interaction with napabucasin were conducted *in vitro* and *in vivo*.

## 2 Materials and methods

### 2.1 Chemicals and reagents

Arbidol and its metabolite, M6-1, were obtained from Shanghai Canspec Scientific Instruments Co., Ltd. (Shanghai, China). Lopinavir, used as the internal standard (IS), and napabucasin were also purchased from Shanghai Canspec Scientific Instruments Co., Ltd. (Shanghai, China). Analytical instruments were filled with acetonitrile and methanol that were purchased from Merck (Darmstadt, Germany). Human liver microsomes (HLMs) and CYP3A4.1 were obtained from iPhase Pharmaceutical Services Co., Ltd. (Beijing, China). Reduced nicotinamide adenine dinucleotide phosphate (NADPH) was obtained from Roche Pharmaceutical Ltd. (Basel, Switzerland). A Milli-Q Ultrapure Water System (Millipore, Bedford, United States) was used to produce purified water.

### 2.2 Instruments and UPLC-MS/MS

Quantitative analysis was performed using a combination of the Waters Xevo TQ-S triple quadrupole tandem mass spectrometer and a Waters ACQUITY UPLC I-Class system (Milford, MA, United States). The instrument was equipped with an automatic sample injection program to achieve fully automatic sample filling and accurate detection. The ACQUITY BEH C18 column (2.1 mm × 50 mm, 1.7 μm; Milford, MA, United States) used for accurate separation of the analytes in the sample at 40°C with acetonitrile (solvent A) and 0.1% formic acid (solvent B) as mobile phases. The gradient elution program was conducted at a flow rate of 0.4 mL/min as follows: 0–0.5 min (10% A+ 90% B), 0.5–1.0 min (10% A increase to 90% A), 1.0–1.4 min (maintain 90% A+ 10% B), 1.4–1.5 min (90% A decrease to 10% A), and 1.5–2.0 min (10% A+ 90% B). Triple quadrupole mass spectrometers were equipped with electrospray ionization (ESI) sources, and multiple reaction monitoring (MRM) modes were used for quantification. Depending on the ion, we selected different ion monitoring voltage conditions, and the results are shown in Table 1.

**TABLE 1:** Analytical parameters of arbidol, M6-1, and internal standard (IS).

Analyte	Ionization mode	Parent (*m/z*)	Daughter (*m/z*)	Cone (V)	Collision (V)
Arbidol	ESI ^+^	478.80	433.80	20	16
M6-1	ESI ^+^	494.83	369.87	10	12
IS	ESI ^+^	629.61	182.98	10	15

### 2.3 Preparation of rat liver microsomes (RLMs)

Rat livers were weighed and homogenized with cold 0.01 mM phosphate-buffered saline (PBS, pH 7.4) containing 0.25 mM sucrose. The homogenates were centrifuged at 11,000 rpm at 4°C for 15 min, and the supernatants were centrifuged repeatedly. Then, the supernatants were transferred to new tubes, centrifuged at 75,600 x g for 2 h, the supernatants were discarded, and three or four times the volume of cold 0.01 mM phosphate-buffered saline was added to the precipitate for homogenization. Finally, the protein concentrations were determined using the Pierce™ BCA Protein Assay Kit (Thermo Scientific) (He et al., 2018).

### 2.4 *In vitro* DDI studies in RLM, HLM, and CYP3A4.1

The 200 μL incubation system was as follows: 0.3 mg/mL RLM (0.3 mg/mL HLM), 1 mM NADPH, pH 7.4 phosphate buffer, and arbidol. Arbidol was used in a range of concentrations (1, 5, 10, 20, 50, 100, and 200 μM) to determine K_m_ (Michaelis constant) in RLM. In HLM, a series of concentrations (0.1, 0.5, 2, 5, 10, 15 and 20 μM) of arbidol were used to determine K_m_. In CYP3A4.1, the following concentrations of arbidol were used to determine K_m:_ 1, 10, 20, 50, 150, 200, 250 μM. Napabucasin was established at concentrations 0.01, 0.1, 1, 10, 25, 50, and 100 μM to determine its IC_50_ (half-maximal inhibitory concentration) *versus* arbidol (4.66, 2.63, and 28.39 μM in RLM, HLM, and CYP3A4.1, respectively, according to their K_m_ values). In addition, the changes in Michaelis–Menten curves in the presence of napabucasin were investigated in RLM and HLM. To study the type of inhibition mechanism, we used the Lineweaver–Burk plot analysis and inhibition constant (Ki and αKi) calculation, where drug concentrations were set as follows: 1.17, 2.33, 4.66, and 9.32 μM for arbidol and 0, 1.13, 2.25, and 4.50 μM for napabucasin in RLM. In HLM, the concentrations of arbidol were 0.66, 1.32, 2.63, and 5.26 μM, and those of napabucasin were 0, 0.98, 1.95, and 3.90 μM. The components in the incubation system were pre-incubated at 37°C for 5 min. After adding NADPH, the reaction was initiated and incubated at 37°C for 30 min. After the stop reaction at −80°C, 400 μL of acetonitrile and 20 μL of IS (lopinavir 200 ng/mL) were added for post-treatment, vortexed for 2 min, and centrifuged at 13,000 rpm for 10 min. Finally, the supernatant was used for quantitative analysis using UPLC-MS/MS.

### 2.5 *In vivo* pharmacokinetic experiments

Sprague–Dawley rats (200 ± 20 g, approximately 6 weeks) were supplied by The First Affiliated Hospital of Wenzhou Medical University and used for the pharmacokinetic experiments. Except for fasting 12 h before the start of pharmacokinetic experiments, rats were fed on a standard rodent diet and kept in a 12-h light–dark cycle environment at 20°C–26°C and 55 ± 15% relative humidity.

Rats were randomly divided into two groups of five each. Arbidol and napabucasin were soluble in 0.5% carboxymethylcellulose sodium solution (CMC-Na). The treatment group was given 48 m/kg napabucasin by gavage, and the control group was given 0.5% CMC-Na solution of the same volume. Thirty minutes after the treatment, each rat was given a single administration of 20 mg/kg of arbidol. Blood samples were obtained from the caudal veins at different times (0.5, 0.75, 1, 1.5, 2, 4, 6, 8, 12, and 24 h). A volume of 10 μL of IS (200 ng/mL) and 300 μL of acetonitrile was added to 100 μL of plasma and mixed in the Eppendorf tube. The UPLC-MS/MS analysis of the supernatant was conducted after centrifuging the mixture at 13,000 rpm for 10 min.

### 2.6 Data analysis

Calculations of IC_50_ values and enzyme kinetic parameters were carried out using GraphPad Prism 6.0 (GraphPad software Inc., CA, United States). The key pharmacokinetic parameters were analyzed using DAS software (Version 3.0 software, Shanghai University of Traditional Chinese Medicine, China). Means ± standard deviations (SD) were calculated for each experiment.

## 3 Results

### 3.1 Method validation

**FIGURE 1:**
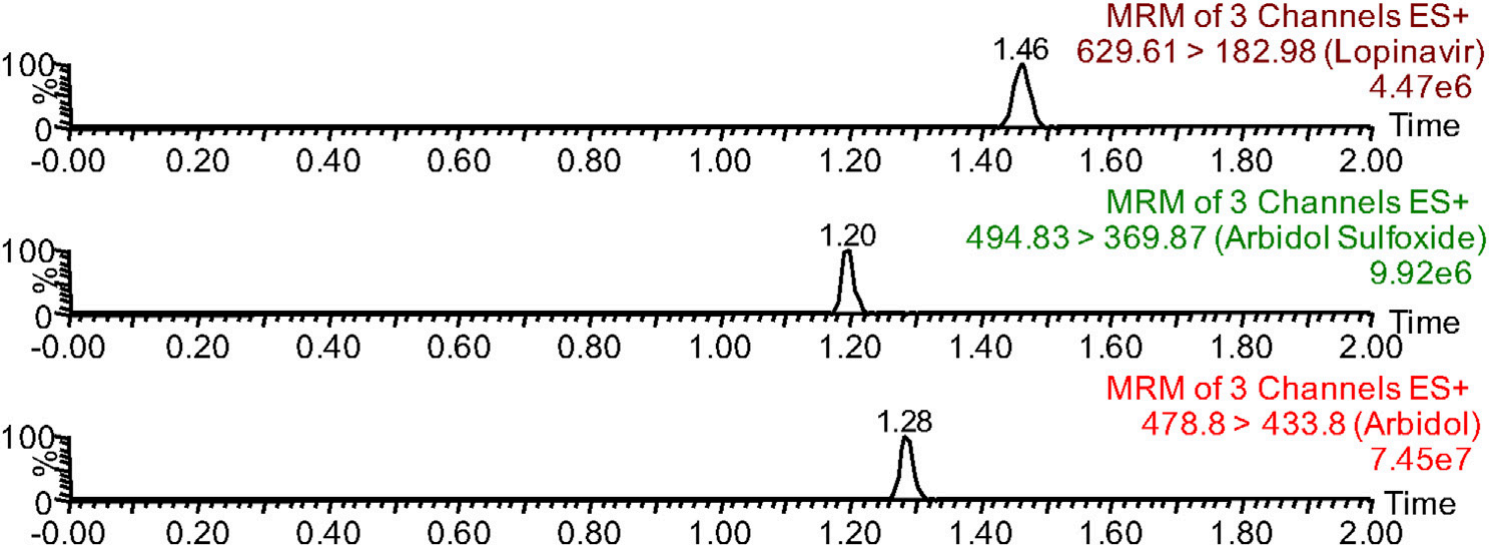
Chromatographic separation time of arbidol, arbidol sulfoxide (M6-1), and lopinavir (IS) in UPLC-MS/MS.

The methodologies were compliant with the standards of the EMA and FDA for bioanalytical methods (U.S. Department of Health and Human Services, Food and Drug Administration, FDA, and Center for Drug Evaluation and Research, CDER, 2018; European Medicines Agency, 2011). In this paper, a methodology was presented that enables the quantitative analysis of arbidol in plasma along with its main metabolite, M6-1. Arbidol, lopinavir, and M6-1 could be detected separately in UPLC-MS/MS without endogenous interference, and their retention times were 1.28, 1.46, and 1.20 min, respectively (Figure 1). The concentration ranges of arbidol and M6-1 for standard curves were 1–200 ng/mL and 1–100 ng/mL, respectively. Their correlation coefficients were *r* ^2^ > 0.99. Both arbidol and M6-1 had acceptable precision and accuracy with a low limit of quantitation (LLOQ) of 1.0 ng/mL.

### 3.2 Effects of napabucasin on the metabolism of arbidol *in vitro*

**FIGURE 2:**
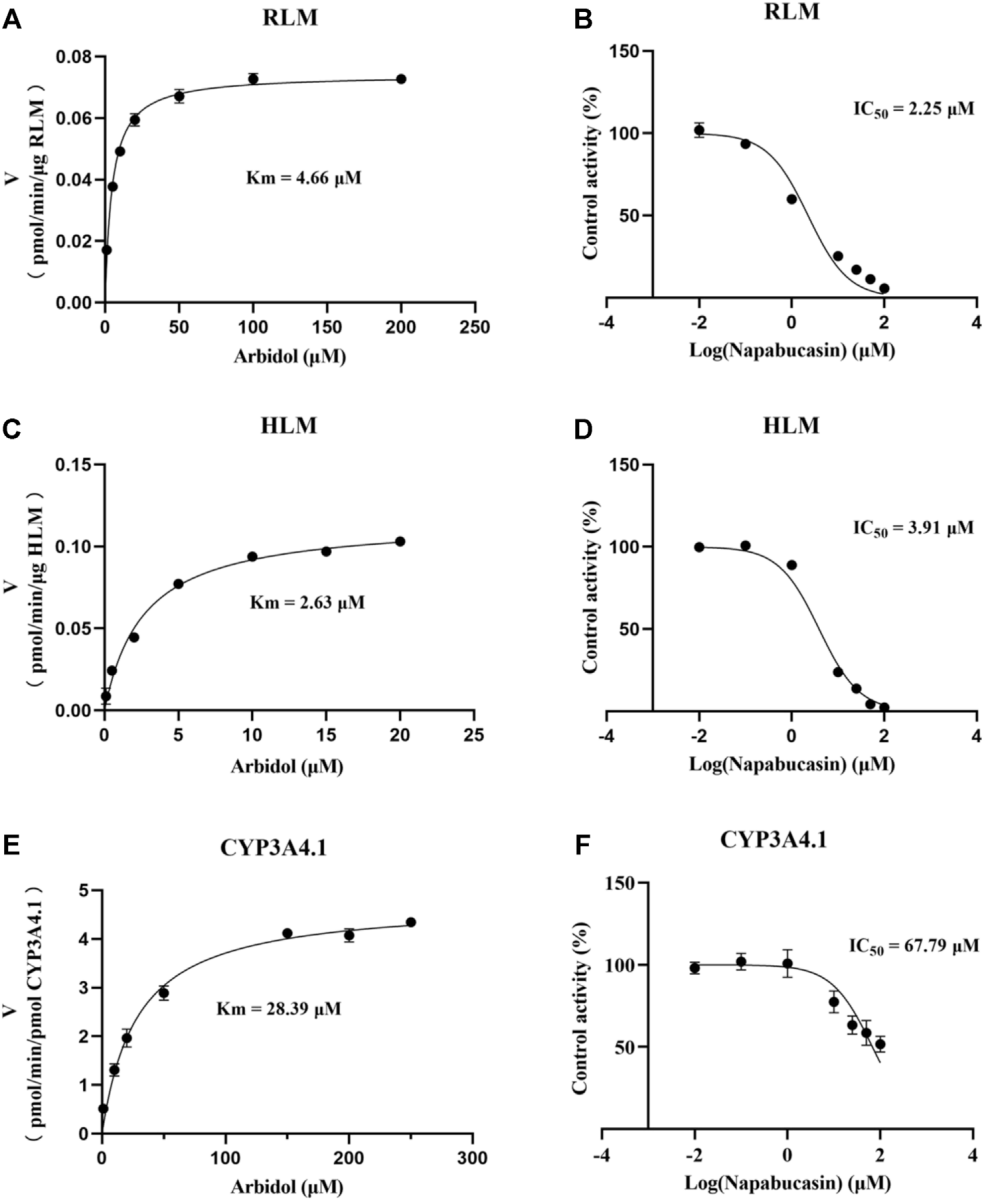
Michaelis constant of arbidol and half-maximal inhibitory concentration (IC_50_) curve of napabucasin on arbidol in RLM **(A, B)**, HLM **(C, D)**, and CYP3A4.1 **(E, F)**, respectively (mean ± SD).

**FIGURE 3:**
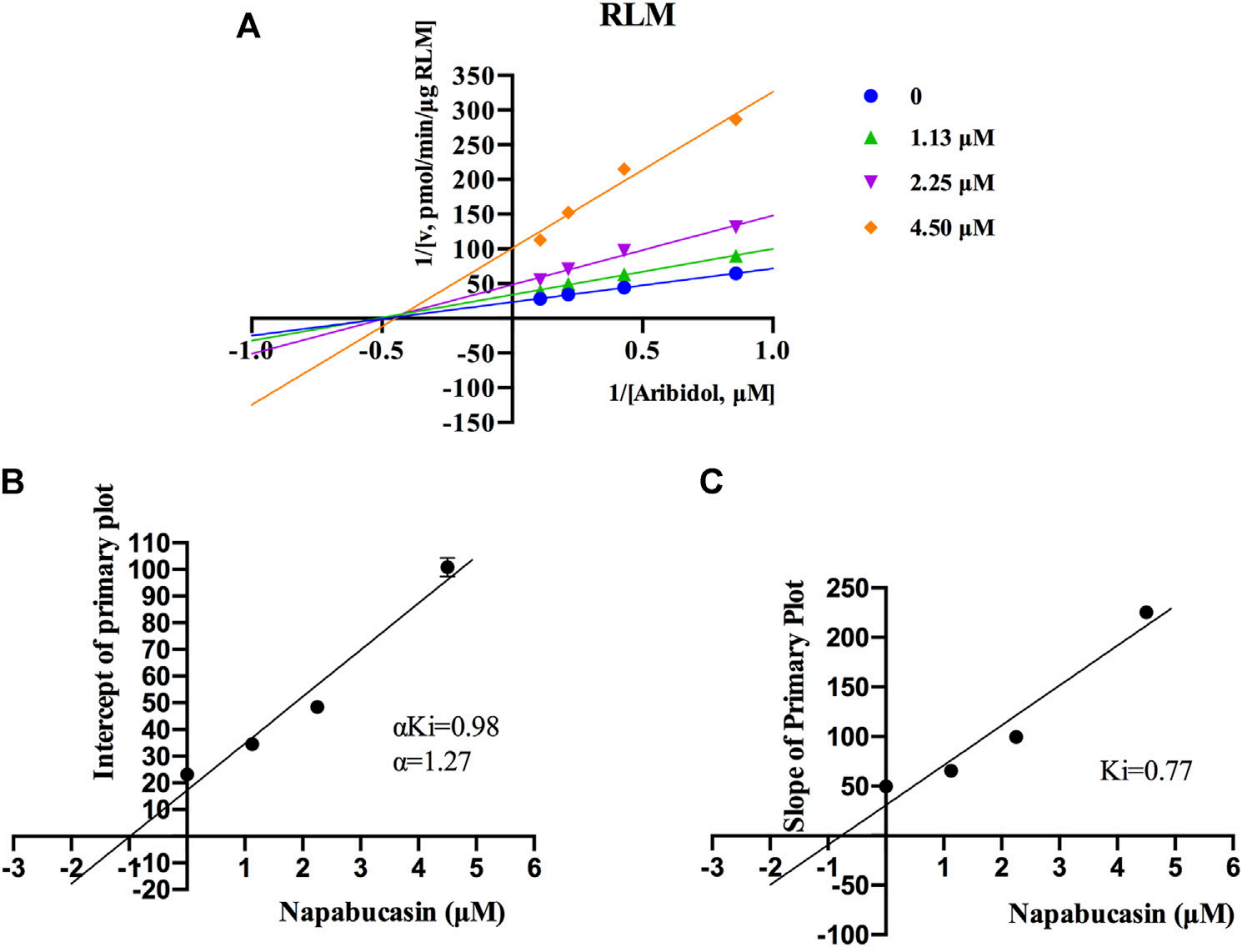
Primary Lineweaver–Burk plots of napabucasin inhibition on arbidol in RLM **(A)**; intercept of the primary plot of napabucasin **(B)**; slope of the primary plot of napabucasin **(C)** (mean ± SD).

**FIGURE 4:**
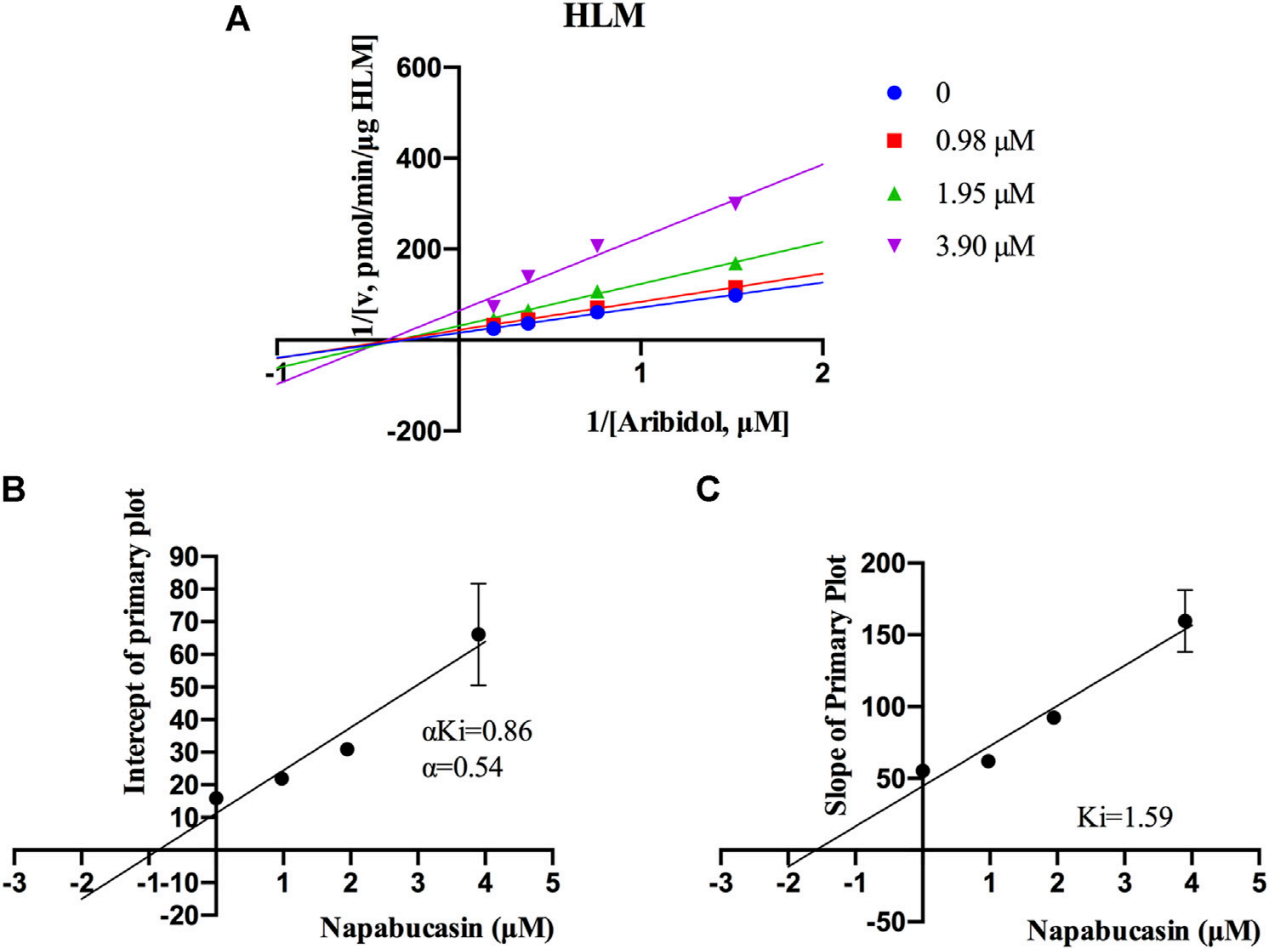
Primary Lineweaver–Burk plots of napabucasin inhibition on arbidol in HLM **(A)**; intercept of the primary plot of napabucasin **(B)**; slope of the primary plot of napabucasin **(C)** (Mean ± SD).

Using the aforementioned incubation system, in RLM, the K_m_ of arbidol was 4.66 μM, the IC_50_ of napabucasin for arbidol was 2.25 μM, and the metabolic rate of arbidol was decreased to 3.73% in the presence of 100 μM napabucasin. In HLM, the K_m_ of arbidol was 2.63 μM and the IC_50_ of napabucasin for arbidol was 3.91 μM (Figure 2). In addition, the Michaelis–Menten parameters for napabucasin inhibition of arbidol had remarkable changes (Supplementary Figures S1, S2), where V_max_ and CL_int_ were decreased to 43.1% and 18.2% in RLM and 59.0% and 50.0% in HLM, respectively. Among the types of inhibition mechanisms, arbidol inhibited by napabucasin in RLM was non-competitive inhibition because the Lineweaver–Burk plot showed that the family of straight lines intersected on the negative semi-axis of y, and the parameters Ki and αKi (*α* = 1.27) were 0.77 and 0.98 μM, respectively (Figure 3). In HLM, the inhibition type of napabucasin on arbidol was mixed inhibition, as indicated by the Lineweaver–Burk plot, which showed that a family of straight lines intersected in the third quadrant, and the parameters Ki and αKi (*α* = 0.54) were 1.59 and 0.86 μM, respectively (Figure 4). Moreover, in CYP3A4.1, the K_m_ and IC_50_ values of the assayed arbidol were 28.39 and 67.79 μM, respectively (Figure 2). Due to its excessive size (>10 μM), the mechanism of inhibition of napabucasin on arbidol in CYP3A4.1 was not explored further.

### 3.3 Effect of napabucasin on the metabolism of arbidol *in vivo*

**FIGURE 5:**
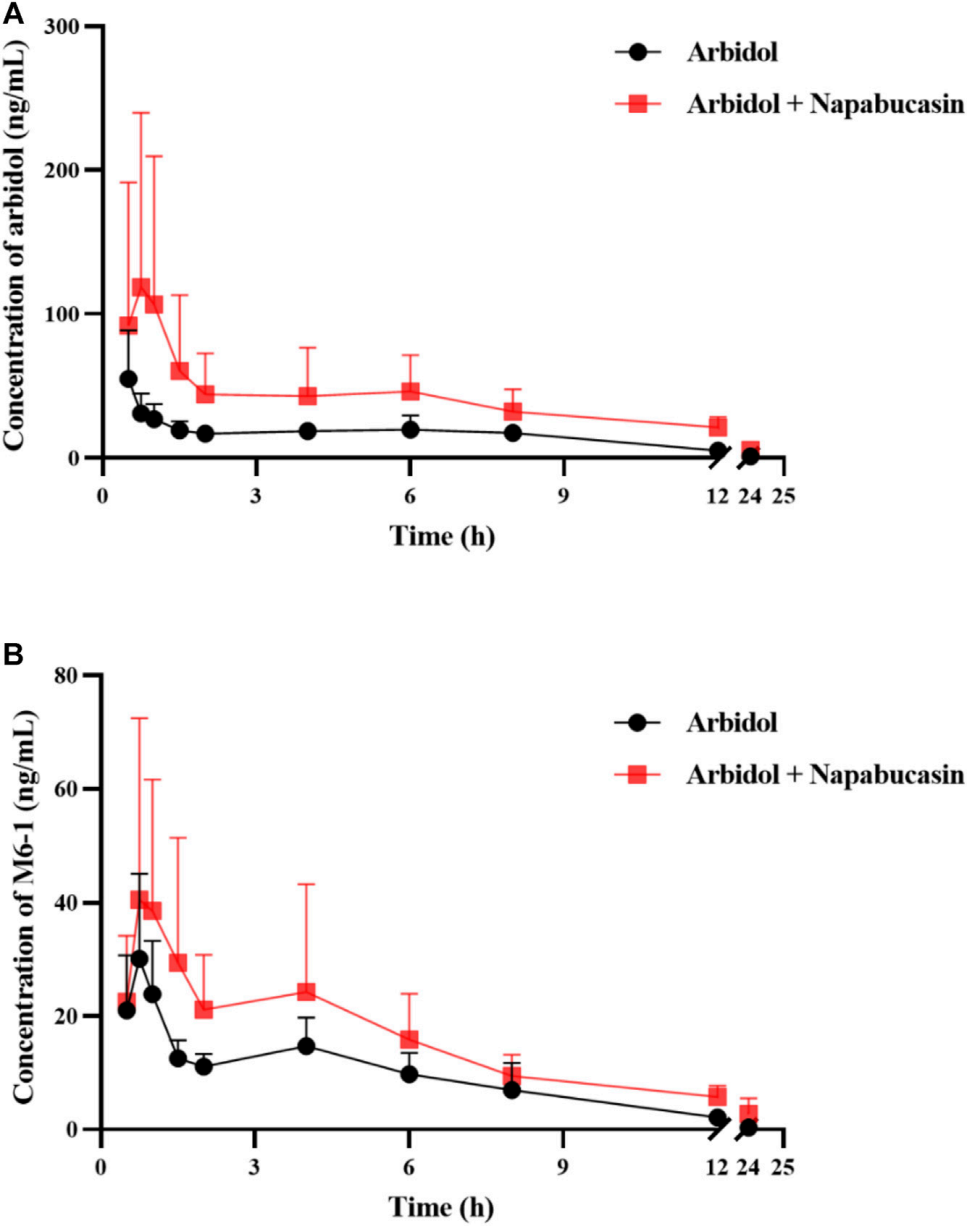
Average plasma concentration–time curve of arbidol **(A)** and its metabolite M6-1 **(B)** in the control group (arbidol alone) and the treatment group (arbidol with napabucasin) (*n* = 5).

The average plasma concentration–time curve of arbidol and its metabolite M6-1 in the control group (arbidol alone) and the treatment group (arbidol with napabucasin) is shown in Figure 5. The important pharmacokinetic parameters in rats are shown in Tables 2, 3. Compared to the control group, the AUC_(0-t)_ of arbidol in rats in the treatment group was increased by 1.85-fold, and the AUC_(0-∞)_ was increased by 1.59-fold. In addition, the parameters CL_z_/F were reduced by 58.0% (Table 2), which indicated that the metabolism of arbidol was inhibited by napabucasin. We measured the levels of the main metabolite M6-1 in the plasma of rats and found that M6-1 levels in the treatment group were similarly affected compared to the control group. The AUC_(0-t)_ of M6-1 in rats from the treatment group was increased by 78.2%, and AUC_(0-∞)_ was increased by 93.1%. Interestingly, the parameters CL_z_/F were reduced by 47.1% (Table 3). Based on the pharmacokinetic results, napabucasin inhibited arbidol metabolism in rats.

**TABLE 2:** Main pharmacokinetic parameters of arbidol in the control group (arbidol alone) and the treatment group (arbidol with napabucasin) of rats (*n* = 5; mean ± SD).

Parameter	Arbidol	Arbidol + napabucasin
AUC_(0-t)_ (ng/mL*h)	235.99 ± 46.18	671.47 ± 297.18^*^
AUC_(0-∞)_ (ng/mL*h)	277.60 ± 15.00	719.51 ± 276.83^**^
t_1/2z_ (h)	5.87 ± 2.54	6.27 ± 2.64
T_max_ (h)	0.60 ± 0.14	1.05 ± 0.57
CL_z_/F (L/h/kg)	72.21 ± 3.75	30.32 ± 8.34^***^
C_max_ (ng/mL)	56.25 ± 32.61	143.33 ± 126.28

*p* < 0.05, *p* < 0.01, and *p* < 0.001, compared with the arbidol alone. AUC, area under the plasma concentration–time curve; t_1/2_z, elimination half time; T_max_, peak time; CL_z_/F, plasma clearance; and C_max_, maximum plasma concentration.

**TABLE 3:** Main pharmacokinetic parameters of M6-1 in the control group (arbidol alone) and the treatment group (arbidol with napabucasin) of rats (*n* = 5; mean ± SD).

Parameter	Arbidol	Arbidol + napabucasin
AUC_(0-t)_ (ng/mL*h)	139.67 ± 31.87	248.96 ± 81.16^*^
AUC_(0-∞)_ (ng/mL*h)	134.38 ± 30.92	259.55 ± 80.21^*^
t_1/2z_ (h)	3.77 ± 1.23	5.12 ± 1.94
T_max_ (h)	1.00 ± 0.56	1.25 ± 0.50
CL_z_/F (L/h/kg)	156.64 ± 42.82	82.87 ± 24.17^*^
C_max_ (ng/mL)	31.08 ± 13.11	48.52 ± 28.46

*p* < 0.05, compared with the arbidol alone. AUC, area under the plasma concentration-time curve; t_1/2_z, elimination half time; T_max_, peak time; CL_z_/F, plasma clearance; and C_max_: maximum plasma concentration.

## 4 Discussion

Arbidol is a popular drug for the treatment of COVID-19, and its pharmacokinetic metabolism in the body is gradually attracting attention (Deng et al., 2013; Hu et al., 2021; Yu et al., 2022). In the context of the gradual implementation of individualized drug application, we established a rapid and effective detection method to detect the levels of arbidol and its metabolite using UPLC-MS/MS.

The chromatogram analysis results of UPLC-MS/MS showed that there was no interference between arbidol, M6-1, and IS during the detection process. When 1 < IC_50_ < 10 μM, it was considered medium inhibitory efficiency (Jin et al., 2015). The metabolic study of arbidol in the incubation system we established found that the IC_50_ of napabucasin for arbidol was IC_50_ = 2.25 μM < 10 μM in RLM and IC_50_ = 3.91 μM < 10 μM in HLM. This indicated that napabucasin exhibited medium inhibition on the metabolism of arbidol in RLM and HLM. Additionally, napabucasin inhibited the metabolism of arbidol to 3.73% in RLM. Among the many anti-tumor drugs, we also found that the metabolic inhibition rate of olmutinib, adagrasib, and sunitinib on arbidol was also below 10% (this part of the data has not yet been published). To prevent adverse reactions, it is recommended to pay attention to DDI when using chemotherapy drugs with arbidol to treat diseases in the clinic. Arbidol inhibition studies with napabucasin showed that there were differences between species *in vitro*. The type of inhibition mechanism was non-competitive inhibition in RLM, while the inhibition type was mixed inhibition in HLM. In addition, in HLM, Ki and αKi (*α* = 0.54) were 1.59 and 0.86 μM, respectively. However, the mechanism in CYP3A4.1 was not investigated due to its excessive IC_50_.

Combination medications are used to treat critical patients with COVID-19 and cancer (Deng et al., 2020; Amani et al., 2021). Arbidol is mainly metabolized by the CYP3A4 isoenzyme in humans, so theoretically inducers or inhibitors of CYP3A4 will affect the metabolism of arbidol. LPV/r as protease inhibitors had a significant interaction with arbidol, which increased the C_max_ and AUC_(0-∞)_ of both drugs (Huang et al., 2021). In this experiment, napabucasin is found to be a bioactivator of quinone oxidoreductase 1 that generates reactive oxygen species (Chang et al., 2019; Froeling et al., 2019). Clinically, it is mainly used for the combination of gastric cancer and metastatic colon cancer in treating diseases (Li et al., 2015). Previous studies showed that *in vitro*, napabucasin is an inhibitor of the CYP2C9, CYP2C19, and CYP3A isozymes; *in vivo* DDI experimental clinical studies investigating the effect of napabucasin on the seven main cytochrome enzymes in the human body found that it may only cause moderate inhibition of CYP2B6 and has no induction or inhibitory effect on CYP1A2, 2C8, 2C9, 2C19, 2D6, 3A, or the BCRP/OAT3 (Dai et al., 2021). According to our results, the *in vivo* pharmacokinetic results of our study in rats are not consistent with this result, which showed that the combination of napabucasin and arbidol increased the concentration of arbidol in rats. Pharmacokinetic parameters were statistically significant, including AUC_(0-t)_ and AUC_(0-∞)_ (Table 2). Under the influence of napabucasin, the CL_z/_F of both arbidol and its main metabolite M6-1 had been slowed compared to the drug alone. The slowdown of M6-1 metabolism may further affect the metabolism of arbidol *in vivo*, so future studies should pay attention to the plasma concentration of the metabolite.

The aforementioned results showed that napabucasin inhibited arbidol metabolism *in vivo* and *in vitro*. The differences in inhibitory effects in RLM and HLM may come from differences between species, as the amount of CYP3A4 expressed was not the same between species. Previous studies reported that differences in sexes can be observed in arbidol metabolism, but unfortunately, this experiment did not compare the metabolic differences of napabucasin and arbidol between rat sexes. A future study in clinics should investigate the effect of napabucasin on arbidol metabolism in humans and whether gender affects the interaction between the two drugs.

## 5 Conclusion

The metabolism of arbidol in RLM and HLM was moderately inhibited by napabucasin *in vitro*, except for CYP3A4.1, with weak inhibition. Its inhibition mechanism was non-competitive in RLM and mixed inhibition (competitive and non-competitive) in HLM. Moreover, in rat pharmacokinetic studies, napabucasin inhibited the metabolism of arbidol and its main metabolite M6-1 in a subtle way. In future studies, more attention should be paid to the combination of these two drugs, and the DDI in humans needs to be further explored.

## Data Availability

The original contributions presented in the study are included in the article/Supplementary Material; further inquiries can be directed to the corresponding author.

## References

[B1] Amani B. Amani B. Zareei S. Zareei M. (2021). Efficacy and safety of arbidol (umifenovir) in patients with COVID-19: a systematic review and meta-analysis. Immun. Inflamm. Dis. 9 (4), 1197–1208. 10.1002/iid3.502 34347937 PMC8426686

[B2] Boriskin Y. Leneva I. Pécheur E. Polyak S. J. C. (2008). Arbidol: a broad-spectrum antiviral compound that blocks viral fusion. viral fusion 15 (10), 997–1005. 10.2174/092986708784049658 18393857

[B3] Brito-Dellan N. Tsoukalas N. Font C. (2022). Thrombosis, cancer, and COVID-19. Support Care Cancer 30 (10), 8491–8500. 10.1007/s00520-022-07098-z 35567609 PMC9106567

[B4] Cai Q. Huang D. Yu H. Zhu Z. Xia Z. Su Y. (2020). COVID-19: abnormal liver function tests. J. Hepatol. 73 (3), 566–574. 10.1016/j.jhep.2020.04.006 32298767 PMC7194951

[B5] Chang A. Hsu E. Patel J. Li Y. Zhang M. Iguchi H. (2019). Evaluation of tumor cell-tumor microenvironment component interactions as potential predictors of patient response to napabucasin. Mol. Cancer Res. 17 (7), 1429–1434. 10.1158/1541-7786.MCR-18-1242 31043490

[B6] Chen L. Wu Y. Gao X. Sun Y. J. (2020). Topical arbidol for the treatment of verruca plantar: a case report. Dermatol Ther. 33 (6), e14497. 10.1111/dth.14497 33145939

[B7] Dai X. Karol M. Hitron M. Hard M. Goulet M. McLaughlin C. (2021). Napabucasin drug-drug interaction potential, safety, tolerability, and pharmacokinetics following oral dosing in healthy adult volunteers. Orig. Manuscr. 10 (8), 824–839. 10.1002/cpdd.961 PMC8453567 34107166

[B8] Deng L. Li C. Zeng Q. Liu X. Li X. Zhang H. (2020). Arbidol combined with LPV/r versus LPV/r alone against Corona Virus Disease 2019: a retrospective cohort study. Virus Dis. 2019 A Retrosp. cohort study 81 (1), e1–e5. 10.1016/j.jinf.2020.03.002 PMC7156152 32171872

[B9] Deng P. Zhong D. Yu K. Zhang Y. Wang T. Chen X. (2013). Pharmacokinetics, metabolism, and excretion of the antiviral drug arbidol in humans. Antimicrob. Agents Chemother. 57 (4), 1743–1755. 10.1128/AAC.02282-12 23357765 PMC3623363

[B10] European Medicines Agency (2011). Guideline on bioanalytical method validation, committee for medicinal products for human use (CHMP) (Accessed February 1, 2021). Available at: https://www.ema.europa.eu/en/bioanalytical-method-validation.

[B11] Froeling F. Swamynathan M. Deschênes A. Chio I. Brosnan E. Yao M. (2019). Bioactivation of napabucasin triggers reactive oxygen species-mediated cancer cell death. Clin. Cancer Res. 25 (23), 7162–7174. 10.1158/1078-0432.CCR-19-0302 31527169 PMC6891204

[B12] He J. Fang P. Zheng X. Wang C. Liu T. Zhang B. (2018). Inhibitory effect of celecoxib on agomelatine metabolism *in vitro* and *in vivo* . Drug Des. Devel Ther. 12, 513–519. 10.2147/DDDT.S160316 PMC5849912 29563776

[B13] Hu Y. Zuo M. Wang X. Wang R. Li L. Lu X. (2021). Pharmacokinetic interactions between the potential COVID-19 treatment drugs lopinavir/ritonavir and arbidol in rats. J. Zhejiang Univ. Sci. B 22 (7), 599–602. 10.1631/jzus.B2000728 34269012 PMC8284091

[B14] Huang X. Li C. Li C. Li Z. Li X. Liao J. (2021). CYP2C19 genotyping may provide a better treatment strategy when administering escitalopram in Chinese population. Front. Pharmacol. 12, 730461. 10.3389/fphar.2021.730461 34512354 PMC8429954

[B15] Hubbard J. Grothey A. J. D. (2017). Napabucasin: an update on the first-in-class cancer stemness inhibitor. Drugs 77 (10), 1091–1103. 10.1007/s40265-017-0759-4 28573435

[B16] Jin C. He X. Zhang F. He L. Chen J. Wang L. (2015). Inhibitory mechanisms of celastrol on human liver cytochrome P450 1A2, 2C19, 2D6, 2E1 and 3A4. Xenobiotica 45 (7), 571–577. 10.3109/00498254.2014.1003113 25811791

[B17] Kirtipal N. Bharadwaj S. Kang S. J. I. (2020). From SARS to SARS-CoV-2, insights on structure, pathogenicity and immunity aspects of pandemic human coronaviruses. Review 85, 104502. 10.1016/j.meegid.2020.104502 PMC7425554 32798769

[B18] Leneva I. Russell R. Boriskin Y. Hay A. J. (2009). Characteristics of arbidol-resistant mutants of influenza virus: implications for the mechanism of anti-influenza action of arbidol. Antivir. Res. 81 (2), 132–140. 10.1016/j.antiviral.2008.10.009 19028526

[B19] Li Y. Rogoff H. Keates S. Gao Y. Murikipudi S. Mikule K. (2015). Suppression of cancer relapse and metastasis by inhibiting cancer stemness. Res. ARTICLE 112 (6), 1839–1844. 10.1073/pnas.1424171112 PMC4330785 25605917

[B20] Liu C. Zhao Y. Okwan-Duodu D. Basho R. Cui X. (2020). COVID-19 in cancer patients: risk, clinical features, and management. Cancer Biol. Med. 17 (3), 519–527. 10.20892/j.issn.2095-3941.2020.0289 32944387 PMC7476081

[B21] Liu L. Shi F. Tu P. Chen C. Zhang M. Li X. (2021). Arbidol combined with the Chinese medicine Lianhuaqingwen capsule versus arbidol alone in the treatment of COVID-19. Med. Baltim. 100 (4), e24475. 10.1097/MD.0000000000024475 PMC7850685 33530261

[B22] Nguyen H. Salkeld J. Agarwal S. Goodman A. (2021). Compassionate use of REGN-COV2 in the treatment of COVID-19 in a patient with impaired humoral immunity. Clin. Infect. Pract. 12, 100089. 10.1016/j.clinpr.2021.100089 34426799 PMC8373583

[B23] U.S. Department of Health and Human Services Food and Drug Administration (FDA), Center for Drug Evaluation and Research (CDER) (2018). Center for veterinary medicine (CVM), bioanalytical method validation guidance for industry. Cent. Drug Eval. Res. Available: at: https://www.fda.gov/regulatory-information/search-fda-guidance-documents/bioanalytical-method-validation-guidance-industry

[B24] Vellingiri B. Jayaramayya K. Iyer M. Narayanasamy A. Govindasamy V. Giridharan B. (2020). COVID-19: a promising cure for the global panic. Sci. Total Environ. 725, 138277. 10.1016/j.scitotenv.2020.138277 32278175 PMC7128376

[B25] Wang J. Feng H. Li Z. Zhang X. (2019). Napabucasin prevents brain injury in neuronal neonatal rat cells through suppression of apoptosis and inflammation. Microb. Pathog. 128, 337–341. 10.1016/j.micpath.2019.01.019 30659911

[B26] Yu M. Wang D.-C. Li S. Lei Y.-H. Wei J. Huang L.-Y. (2022). Meta-analysis of arbidol versus lopinavir/ritonavir in the treatment of coronavirus disease 2019. J. Med. Virol. 94 (4), 1513–1522. 10.1002/jmv.27481 34837230 PMC9011863

[B27] Zhou X. Hou H. Yang L. Ding G. Wei T. Li C. (2021). Arbidol is associated with increased in-hospital mortality among 109 patients with severe COVID-19: a multicenter, retrospective study. J. Glob. Health 11, 05017. 10.7189/jogh.11.05017 34326998 PMC8284661

