# Supplementary material for: Inhibitory effect of napabucasin on arbidol metabolism and its mechanism research
Source: Front Pharmacol. 2023 Nov 28;14:1292354. doi: 10.3389/fphar.2023.1292354 (PMC10716843; doi:10.3389/fphar.2023.1292354)
Supplement: Supplementary file 1 [file DataSheet1.pdf]

Inhibitory effect of napabucasin on arbidol metabolism and its mechanism research

Supplementary figures: 2

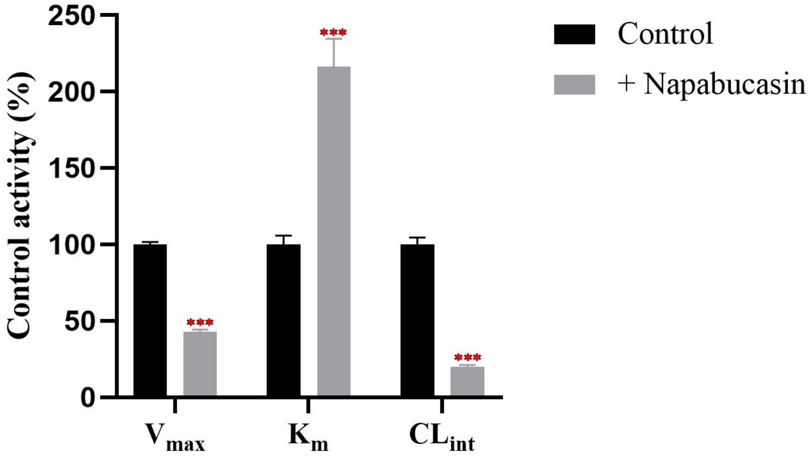

Supplementary Figure S1. The changes of Michaelis-Menten parameters for napabucasin inhibition of arbidol in RLM (n = 3, Mean ± SD). ***P<0.001.

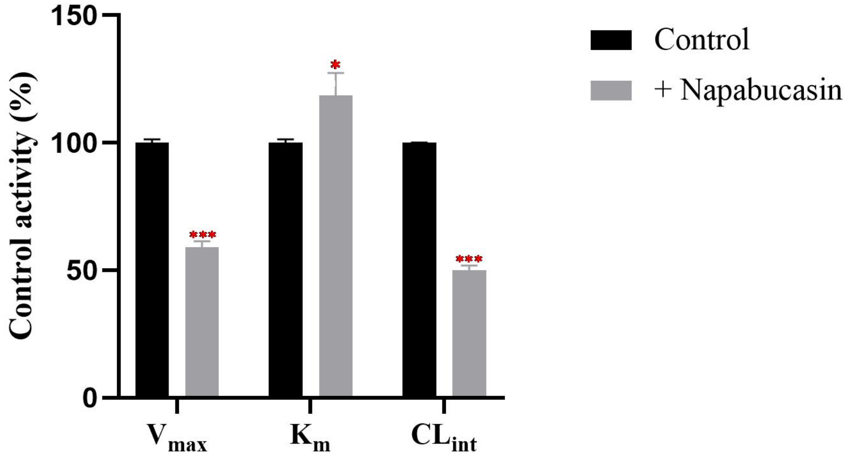

Supplementary Figure S2. The changes of Michaelis-Menten parameters for napabucasin inhibition of arbidol in HLM (n = 3, Mean ± SD). *P<0.05, ***P<0.001.
